# Pars Plana Vitrectomy With Internal Limiting Membrane Peeling for the Treatment of Cystoid Macular Edema in a Patient With Syphilitic Uveitis and HIV Infection

**DOI:** 10.7759/cureus.32865

**Published:** 2022-12-23

**Authors:** Mariella C Pappaterra-Rodriguez, Claudia Amaral, Guillermo A Requejo Figueroa, Sofía C Ayala Rodríguez, Edgar De Jesús Rodríguez, Karla C Alejandro, Armando L Oliver

**Affiliations:** 1 Medicine, Ponce Health Sciences University, Ponce, USA; 2 Ophthalmology, University of Puerto Rico School of Medicine, San Juan, USA

**Keywords:** pars plana vitrectomy, ocular syphilis, internal limiting membrane peeling, hiv, cystoid macular edema

## Abstract

We report a case of cystoid macular edema (CME) secondary to syphilitic uveitis that was successfully treated with pars plana vitrectomy with internal limiting membrane peeling. A 37-year-old male with a history of HIV developed a CME secondary to syphilitic panuveitis. His uveitis resolved following treatment with intravenous penicillin, yet his CME persisted and was refractory to four posterior sub-tenon triamcinolone acetonide injections. A pars plana vitrectomy with internal limiting membrane peeling was performed, resulting in lasting resolution of the CME and the improvement of his visual acuity at the two-month follow-up visit. Pars plana vitrectomy with internal limiting membrane peeling may be a viable alternative for the treatment of CME in patients with syphilitic uveitis. In particular, it may serve as a viable alternative for the treatment of CME in patients with a history of infectious uveitis or other comorbidities, such as HIV infection.

## Introduction

According to the United States Centers for Disease Control and Prevention, syphilitic uveitis should be treated with 10 to 14 days of intravenous penicillin, which is the same treatment used for neurosyphilis [1]. However, patients with syphilitic uveitis may still develop complications despite adequate treatment. One such complication is cystoid macular edema (CME) [2-4]. The presence of syphilis and human immunodeficiency virus (HIV) co-infection can further hinder the treatment of CME [1]. Topical and periocular steroids usually are adequate for treating CME in patients with luetic uveitis, but may not be sufficient to prevent recalcitrant macular edema [1,3].

The medical literature on the use of intraocular steroids to treat macular edema in patients with infectious uveitis is limited to small case series and occasional case reports [2-4]. There are several reports of cytomegalovirus retinitis (CMVR) occurring following intravitreal steroid injections [5-8]. These reports raise the concern that the use of intraocular steroids may trigger CMVR in patients with HIV infection, as specific immune responses to cytomegalovirus may lag for several months following the initiation of combined antiretroviral therapy (cART) [9].

We report a case of an HIV-positive patient with recalcitrant CME secondary to syphilitic panuveitis, in which CME was successfully treated with pars plana vitrectomy with internal limiting membrane peeling. To our knowledge, this is the first such case to be reported in the medical literature. 

## Case presentation

A 37-year-old Hispanic man arrived at the emergency room with vision loss, erythema, and swelling of the left eye, all of which had persisted for one month. A review of systems was positive for a rash on his palms and soles. His past medical history was remarkable for a diagnosis of HIV infection; although his diagnosis of HIV had been made nine years prior, he was not on 200 oculus sinister (OS) when he presented at the emergency room. 

His best-corrected visual acuity was 20/25 oculus dexter (OD) and hand motion OS. Intraocular pressure was normal in both eyes. His pupils were equally reactive to light and accommodation, and no afferent defect was noted. An examination of the right eye was within normal limits. However, a slit-lamp examination of the left eye showed a 2+ conjunctival injection, ciliary flush, non-granulomatous keratic precipitates, posterior synechiae, 4+ anterior chamber cells, and no hypopyon. Left fundus examination revealed 3+ vitritis and peripheral multifocal choroiditis. Following an initial assessment of left panuveitis, a workup was ordered, which revealed a positive venereal disease research laboratory (VDRL) test (1:128) and fluorescent treponemal antibody absorption test, suggesting a diagnosis of ocular syphilis. A cerebrospinal fluid VDRL test, a chest X-ray, and purified protein derivative tests were negative. The patient was treated with topical steroids and a 10-day course of intravenous penicillin. The infectious diseases service was consulted, and cART was started. One month following his initial diagnosis, the patient's visual acuity had improved to 20/50 OS and the topical steroids were tapered and then discontinued. 

Six months following the initial diagnosis, the patient's visual acuity had decreased to 20/200 OS. An examination of the left eye revealed 1+ anterior vitreous cells, atrophic chorioretinal scars, and CME. A subsequent VDRL test, which was ordered to rule out syphilis reinfection, was positive, though at a lower dilution (1:2); therefore, no further treatment with systemic penicillin was advised.

**Figure 1:**
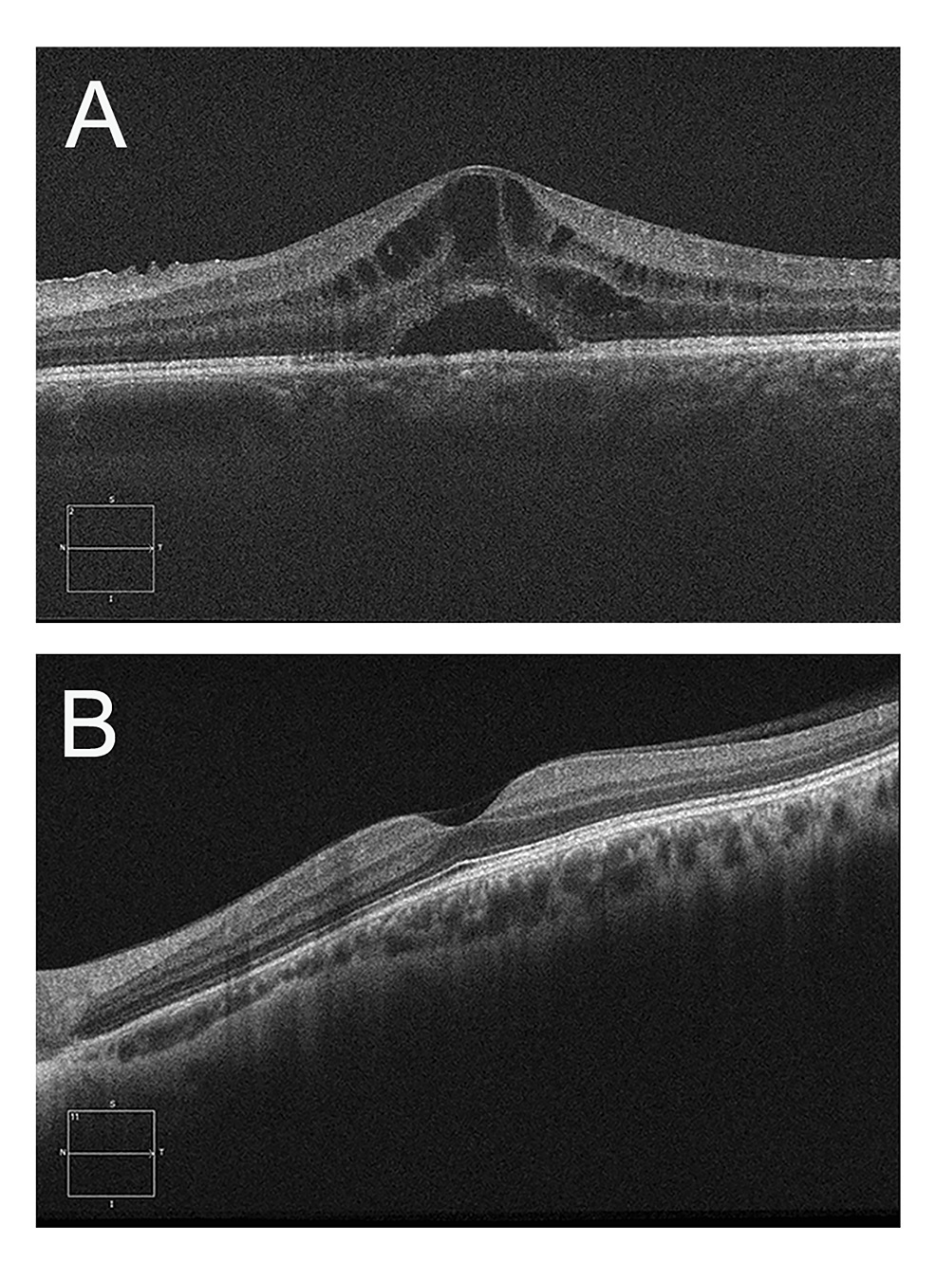
Spectral-domain optical coherence tomography (SD-OCT) of the left eye A: Preoperative images reveal a corrugated internal limiting membrane (ILM) and intraretinal and sub-retinal fluid consistent with a diagnosis of cystoid macular edema (CME) B: The images reveal the resolution of the CME following a pars plana vitrectomy with an ILM peel

During the subsequent 30 months, the patient's compliance with follow-up care was erratic. His treatment with topical steroids resulted in the improvement of the residual vitritis; however, his CME persisted. During this period, he received a total of four posterior sub-tenon triamcinolone acetonide (40 mg/mL) injections for the treatment of his CME. Despite our instructions, the patient was lost to follow-up for serial sub-tenon injections for 18 months and his visual acuity worsened to 20/400, due to the chronic CME (Figure 1, A). This prompted us to consider a surgical alternative for the management of his edema. 

A standard 25-gauge pars plana vitrectomy, with detachment and removal of the posterior hyaloid, was performed, permitting access to the internal limiting membrane (ILM). Indocyanine green solution was used to stain the ILM, an initial flap was created with a curved membrane scraper, and the ILM was removed up to the perifovea. The surgery was performed without complications. Two months after the surgery, his visual acuity improved to 20/70 OS and the CME resolved (as seen above in Figure 1, B).

## Discussion

Pars plana vitrectomy with ILM peeling improves the oxygenation of the inner retina and relieves macular traction, which helps reduce macular edema [10-12]. The ILM peeling has been successfully used to treat macular edema secondary to diabetic retinopathy and retinal vein occlusions [10,11]. A small, retrospective study comparing the use of intravitreal triamcinolone versus vitrectomy with an ILM peel for the treatment of diabetic macular edema suggested that the latter may have the benefit of providing long-lasting resolution of the edema [13]. The favorable outcomes associated with the use of vitrectomy with an ILM peel to treat such causes of macular edema open the possibility of using surgical therapy to treat CME from other etiologies (e.g., uveitis-related CME), particularly in patients whose condition is refractory to other available options.

Our patient had a history of HIV infection and poor compliance with cART, which is why we did not use intraocular steroids. Although there are a few reports of the successful use of intraocular steroids to treat syphilitic uveitis in HIV-positive patients, they should be interpreted cautiously, as no controlled studies have established the safety of steroids in such a treatment [3,4]. Although reports on the reactivation of CMVR following the administration of intraocular steroids are relatively rare, most of these reports have described immunocompetent patients, and HIV-positive patients who are immunocompromised and may be more prone to developing iatrogenic CMVR [5-9]. Additionally, the use of intravitreal triamcinolone has been reported to unmask ocular syphilis, leading to severe panuveitis, occlusive vasculitis, and permanent loss of vision [14]. 

## Conclusions

Our case suggests that a PPV with ILM peeling may be a viable alternative to treat recalcitrant CME in HIV-positive patients with syphilitic uveitis. Long-term controlled studies should be undertaken to explore this alternative further and establish its long-term effectiveness and safety. Although several authors have reported success in their use of intraocular steroids with HIV-positive patients with ocular syphilis, such a treatment may confer a risk of iatrogenic CMVR or cause the unmasking of luetic uveitis. 
